# Supplementary material for: Screening of Antifungal Activity of Essential Oils in Controlling Biocontamination of Historical Papers in Archives
Source: Antibiotics (Basel). 2023 Jan 6;12(1):103. doi: 10.3390/antibiotics12010103 (PMC9854659; doi:10.3390/antibiotics12010103)
Supplement: Supplementary file 1 [file antibiotics-12-00103-s001.zip › antibiotics-2107871-supplementary.pdf]

Supplementary Table S1. Chemical composition of essential oils.

Chemical composition	Essential oil						
		Origanum vulgare		*Mentha piperita*		*Cymbopogon citratus*	
	RI	R.T. min	%	R.T. min	%	R.T. min	%
α-Thujene	924	5.621	1.6	5.650	tr*	/**	/
α-Pinene	931	5.815	0.9	5.814	0.7	/	/
Camphene	945	6.225	0.2	6.225	tr	/	/
Sabinene	970	6.912	8.8	6.908	0.5	/	/
β-Pinene	974	7.014	1.2	7.02	1.1	/	/
3-Octanone	983	7.267	0.1	/	/	/	/
6-Methyl-5-hepten-2-one	987	/	/	/	/	7.398	0.3
Myrcene	988	7.399	2.3	7.407	0.2	7.52	4.8
3-Octanol	991	7.529	0.2	7.524	0.2	/	/
α-Phellandrene	1003	7.872	0.2	7.882	tr	/	/
δ-3-Carene	1009	8.07	0.1	/	/	/	/
α-Terpinene	1014	8.275	2.1	8.277	0.3	/	/
p-Cymene	1022	8.559	11.0	8.557	0.2	/	/
Limonene	1025	8.706	1.2	8.689	1.5	/	/
1,8-Cineole	1028	8.79	0.7	8.781	4.2	/	/
(Z)-β-Ocimene	1033	8.989	3.2	8.994	0.2	/	/
(E)-β-Ocimene	1044	9.374	4.6	9.396	tr	9.555	tr
γ-Terpinene	1055	9.816	19.6	9.787	0.6	/	/
cis-Sabinene hydrate (IPP vs OH)	1063	10.098	0.4	10.09	1.2	/	/
Terpinolene	1086	10.938	0.2	10.939	0.2	/	/
NI	1096	11.328	0.2	11.321	0.1	/	/
Linalool	1097	11.362	1.1	11.372	0.2	11.525	0.7
2- Methyl butyl isovalerate	1105	/	/	11.707	0.1	/	/
1,3,8-p-Menthatriene	1107	/	/	/	/	11.75	tr
NI	1119	12.278	0.1	12.274	0.1		
NI	1120	/	/	/	/	12.204	0.1
allo-Ocimene	1126	12.579	0.6	/	/	/	/
NI	1136	/	/	13.044	0.1	/	/
NI	1142	/	/	13.289	0.1	/	/
Menthone	1150	/	/	13.682	23.8	/	/
NI	1151	/	/	/	/	13.66	0.1
Menthouran	1160	/	/	14.054	7.5	/	/
iso-Menthone		/	/	14.099	3.2	/	/
Borneol	1163	14.146	0.5	/	/	/	/

Z-Isocitral	1166	/	/	/	/	14.301	0.2
Menthol	1171	/	/	14.533	30.3	/	/
Terpinen-4-ol	1174	14.652	1.3	14.684	1.8	/	/
iso-Menthol	1180	/	/	14.921	0.4	/	/
NI	1183	/	/	/	/	14.955	0.1
E-Isocitral	1184	/	/	/	/	15.08	0.4
α-Terpineol	1188	15.237	0.4	15.243	0.4	/	/
NI	1206	16.034	0.1	/	/	/	/
Citronellol	1230	/	/	/	/	17.098	0.2
NI	1233	17.199	0.1	17.257	0.1	/	/
Pulegone	1237	/	/	17.395	7.8	/	/
Carvacrol methyl ether	1242	17.597	1.0	/	/	/	/
Neral	1244	/	/	/	/	17.735	36.9
Piperitone	1252	/	/	18.049	0.2	/	/
NI	1254	18.132	0.1	/	/	/	/
Geraniol	1256	/	/	/	/	18.24	2.9
Geranial	1272	/	/	/	/	19.089	51.5
neo-Menrhyl acetate	1273	/	/	18.986	0.3	/	/
NI	1286	19.523	0.1	/	/	/	/
NI	1287	19.576	0.1	/	/	/	/
Thymol	1291	19.785	3.0	/	/	/	/
Menthyl acetate	1292	/	/	19.838	5.6	/	/
2-Undecanone	1296	/	/	/	/	20.031	0.1
Carvacrol	1301	20.252	15.6	/	/	/	/
iso-Menthyl acetate	1306	/	/	20.468	0.2	/	/
δ-Elemene	1334	/	/	21.732	0.2	/	/
NI	1335	21.751	0.1	/	/	/	/
NI	1340	/	/	/	/	21.976	0.2
NI	1354	/	/	/	/	22.619	0.1
α-Copaene	1373	23.445	0.1	23.438	tr	/	/
NI	1377	/	/	/	/	23.565	0.4
β-Bourbonene	1382	23.838	0.1	23.833	0.3		
Geranyl acetate	1385	/	/	/	/	23.962	0.7
β-Elemene	1389	24.159	0.1	24.148	0.6	/	/
Caryophyllene(E-)	1417	25.34	6.1	25.32	2.8	25.507	tr
β-Copaene	1427	25.739	0.2	25.73	0.1	/	/
α-Humulene	1452	26.769	0.7	26.759	0.1	/	/
(E)-β-Farnesene	1455	/	/	26.902	0.2	/	/
allo-Aromadendrene	1459	27.079	0.1	/	/	/	/
(+)-epi-Bicyclosesquiphellandrene	1461	27.173	0.1	/	/	/	/

γ-Muurolene	1475	27.753	0.1	/	/	/	/
Germacrene D	1480	27.941	2.1	27.928	1.3	/	/
2-Tridecanone	1495	/	/	/	/	28.729	tr
NI	1496	28.524	0.1	/	/	/	/
Bicyclogermacrene	1497	28.582	0.2	28.58	0.5	/	/
α-Muurolene	1499	28.745	0.1	/	/	/	/
(E,E)-α-Farnesene	1507	29.069	2.9	/	/	/	/
γ-Cadinene	1513	29.314	0.3	/	/	/	/
δ-Cadinene	1522	29.7	0.6	29.686	0.1	/	/
α-Cadinene	1536	30.274	0.1	/	/	/	/
NI	1541	30.483	0.1	/	/	/	/
Spathulenol	1575	31.899	0.6	31.891	0.1	/	/
NI	1580	32.034	0.2	32.111	0.1	/	/
Caryophyllene oxide	1581	32.117	0.7	/	/	32.281	tr
NI	1588	/	/	32.458	0.2	/	/
Salvial-4(14)-en-1-one	1592	32.583	0.1	/	/	/	/
Humulene epoxide II	1607	33.17	0.1	/	/	/	/
NI	1609	33.267	0.1	/	/	/	/
NI	1617	/	/	/	/	33.688	0.1
NI	1638	34.379	0.4	/	/	/	/
NI	1644	34.634	0.1	/	/	/	/
NI	1653	34.93	0.1	/	/	/	/
Eudesma-4(15),7-dien-1-beta-ol	1685	36.163	0.1	/	/	/	/
NI	1863	42.664	0.1	/	/	/	/
NI	1901	43.973	0.1	/	/	/	/
NI	2502	62.908	0.1	/	/	/	/
**Total**			**99.9**		**100**		**99.8**
**NI**			**2.3**		**1.0**		**1.1**

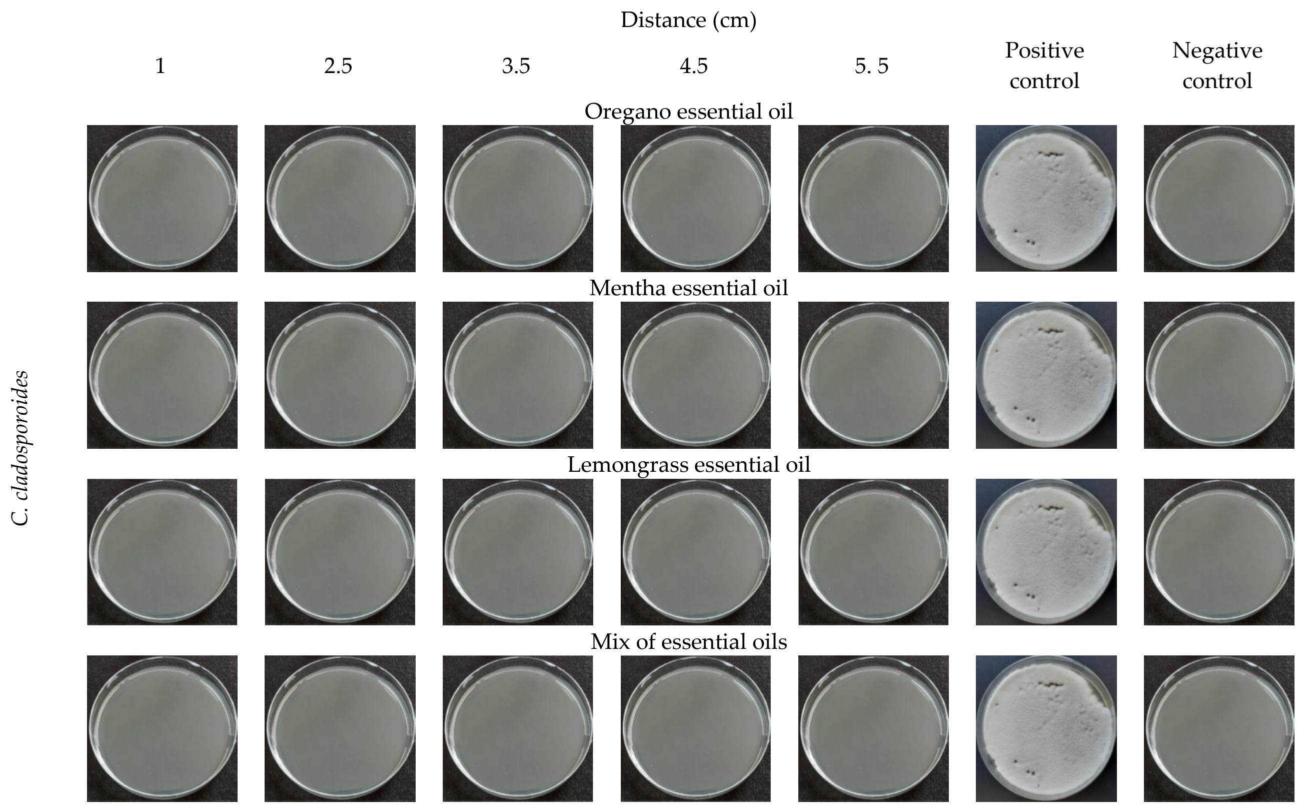

Supplementary Figure S1. EOs efficiency in vapour phase against *C. cladosporoides*

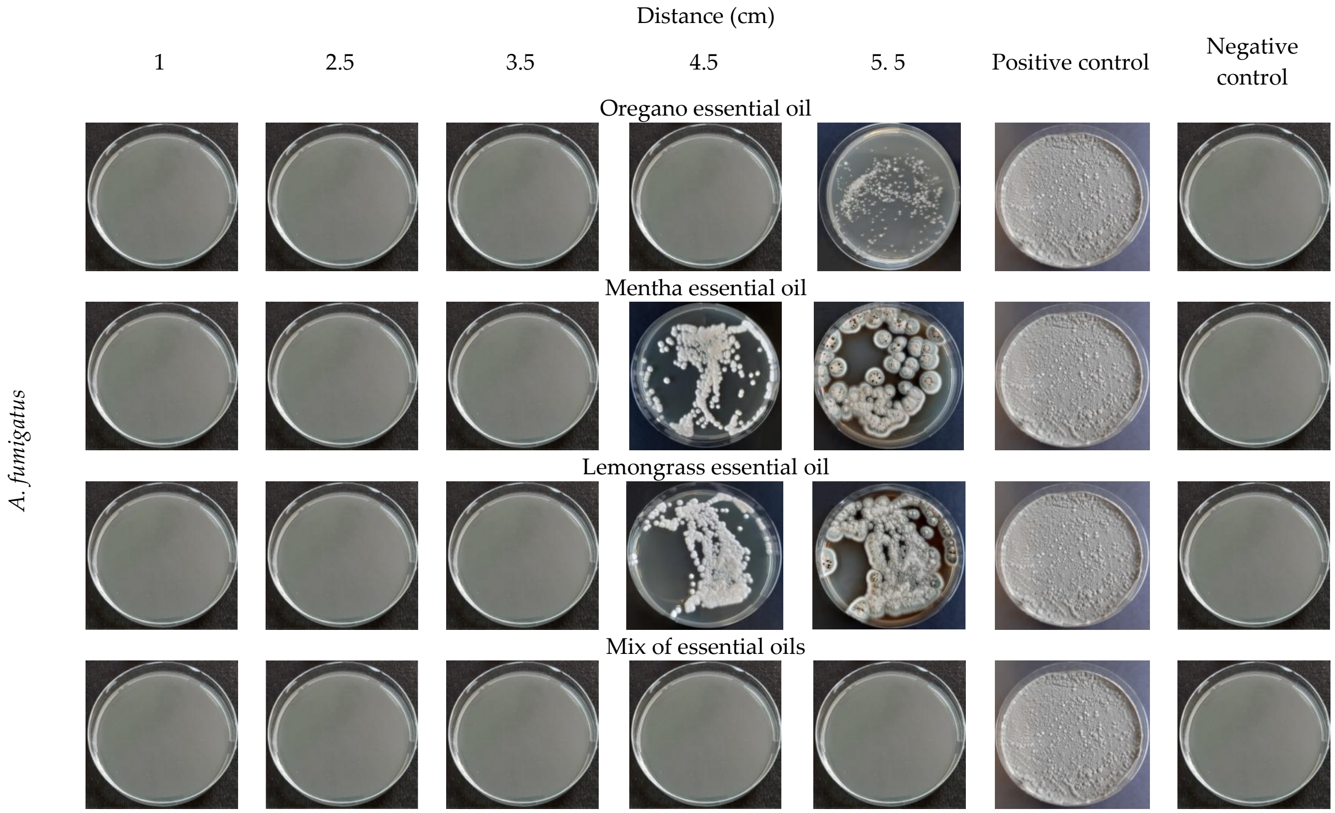

Supplementary Figure S2. EOs efficiency in vapour phase against *A. fumigatus*

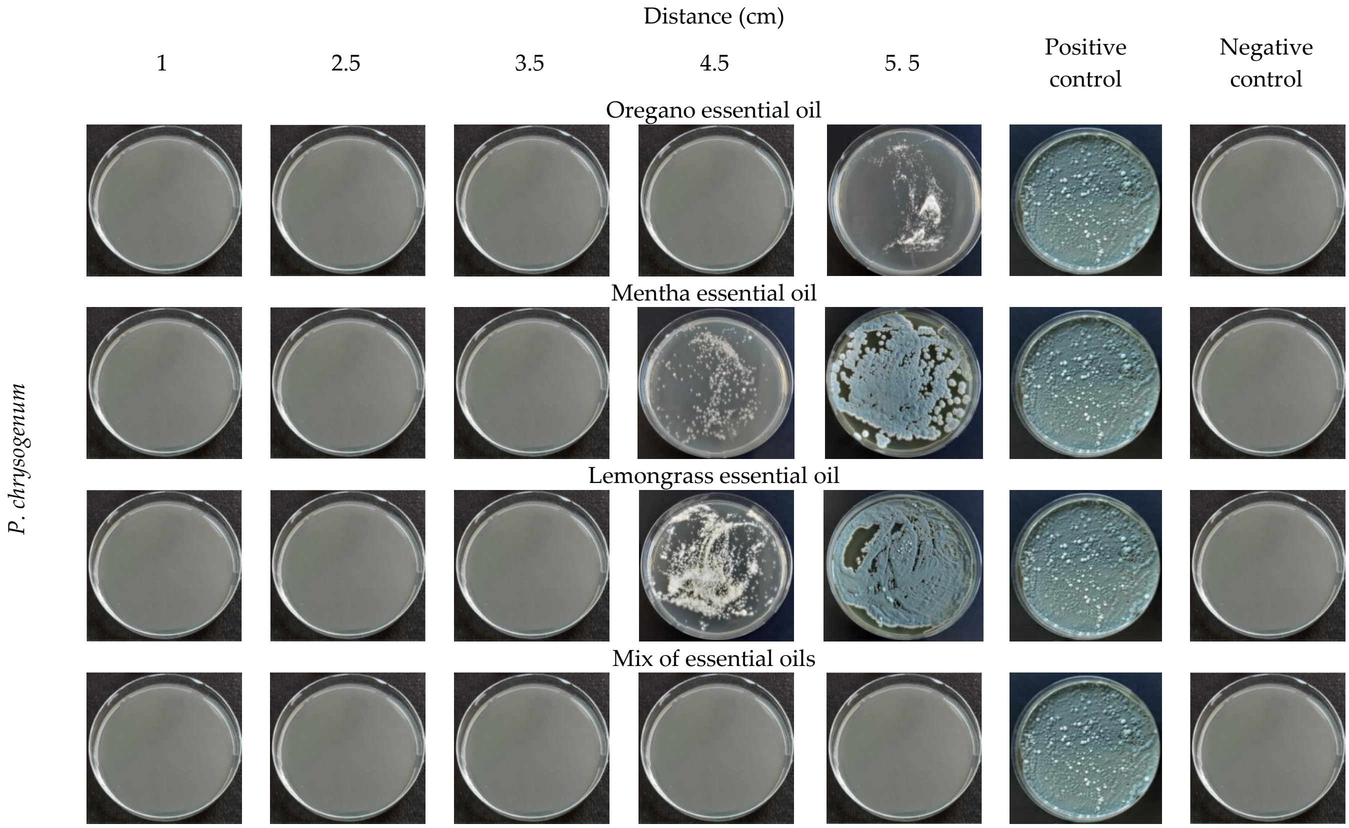

Supplementary Figure S3. EOs efficiency in vapour phase against *P. chrysogenum*
